# Interventions addressing care staff views of older LGBTQ+ people in residential and homecare settings: a scoping review protocol

**DOI:** 10.1136/bmjopen-2024-086497

**Published:** 2024-10-23

**Authors:** Yvonne Moriarty, Paul Willis

**Affiliations:** 1 Centre for Trials Research, Cardiff University, Cardiff, UK; 2 Centre for Adult Social Care Research, Cardiff University, Cardiff, UK

**Keywords:** Education, Nursing Care, Psychosocial Intervention, Review, Sexual and Gender Minorities, Aging

## Abstract

**ABSTRACT:**

**Introduction:**

Previous research has identified that lesbian, gay, bisexual, trans and queer (LGBTQ+) social care service users have concerns and/or negative experiences of their care due to staff views and attitudes about them/their sexual and gender identities. This has resulted in a number of barriers and challenges for the delivery of social care to this population. However, there is a little research relating to what types of evidence-based interventions can help overcome these barriers, enhance knowledge and promote positive attitude change in staff. The objective of this review was to systematically review current literature on interventions targeted at staff views and attitudes of LGBTQ+ older people in care and identify gaps in knowledge to inform a future theory of change and identify potential interventions to address these.

**Methods and analysis:**

This scoping review will be conducted in line with the Joanna Briggs Institute methodology for scoping reviews. We will conduct a comprehensive search of electronic databases (ie, Social Services Abstracts, Social Care online, Sociological Abstracts, PsycINFO, Medline, CINAHL, Scopus, ASSIA and Web of Science) focused on both health and social care literature to identify peer-reviewed literature as well as targeted online searches of potentially relevant grey literature. We will include literature published in the past 20 years (2003–2023) that report interventions to address care staff attitudes of LGBTQ+ populations older than 50 years who are receiving adult social care in a broad range of healthcare, residential or home settings. Citations will be screened by two independent researchers for inclusion and relevant data extracted using a bespoke template. Data will be analysed narratively and summarised to identify gaps in knowledge and aid in developing a theory of change.

**Ethics and dissemination:**

Ethics approval is not required. Findings will be disseminated via publication in a peer-reviewed journal.

**Review registration:**

A priori registration on Open Science Framework: https://osf.io/v76ws

**STRENGTHS AND LIMITATIONS OF THIS STUDY**

- This review will follow rigorous Joanna Briggs Institute methodology for the conduct of scoping reviews and reported in line with the Preferred Reporting Items for systematic reviews and Meta-Analyses Scoping Reviews (PRISMA-ScR) guidelines.
- Databases holding both medical and social care literature will be searched to capture an inclusive definition of adult social care.
- Both peer-reviewed and grey literature will be searched, increasing the potential inclusion of a broad range of relevant literature.
- Quality assessment of the literature and methods will not be conducted as part of the scoping review method.
- The review will be limited to the past 20 years potentially missing relevant citations from the review.

## Introduction

 The world’s ageing population is increasing rapidly, resulting in an increase of people accessing ageing services. The WHO defines old age as those older than 60 years^1^; however, with an ageing population, services are continually adapting to societal needs to include young cohorts to support healthy ageing and transition in services.^2^ Older lesbian, gay, bisexual, trans and queer (LGBTQ+) are an underserved and unrecognised population in adult social care in the UK. The health disparities between older LGBTQ+ adults and their heterosexual counterparts have been internationally recognised^3 4^ with more recent US literature citing disparities in the likelihood of experiencing cognitive impairment.^5^ Within aged care settings, LGBTQ+ service users are frequently reported as ‘invisible’. This is due to several social, cultural and political factors. First, older LGBTQ+ people report low levels of confidence and trust in health and care professionals more broadly.^6 7^ This partly stems from previous experiences of homophobia and transphobia during professional encounters and service interactions as well as experiencing hostile social climates during earlier decades when homosexual relations were criminalised between men. Other forms of social and political exclusion included non-heterosexual and trans identities being pathologised as mental disorders, and estrangement and rejection from family and community networks.^8^ Second, care workers and professionals report LGBTQ+ service users and residents as missing from their services and express feeling unprepared and lacking knowledge and confidence to initiate affirming conversations about non-normative sexuality and gender-based histories and relationships.^9^,^11^ Furthermore, there is resistance to embracing LGBTQ+ residents and identities in care settings with this activity perceived as outside the scope of care provision.^10 12^ Third, LGBTQ+ adults convey concerns about having to retreat back into ‘the closet’ when entering long-term care settings in anticipation of hostile and discriminatory treatment from staff and other residents.^13^ Losing mental capacity over everyday decision-making is another concern that rests on individual anxieties about having one’s autonomy stripped away over how LGBTQ+ identities are expressed and discussed in care settings.^9 14^ Finally, attempts to provide person-centred and equal services to older LGBTQ+ people in care settings are often poorly realised under the discourse of ‘treating everyone the same’ as a method of achieving equality.^11 15^ In reality, this discourse flatlines inequalities between groups of older people and blinkers staff to the unequal life course trajectories that older LGBTQ+ people have experienced. Based on the above research literature, we know there is a need for change in care home staff views, attitudes and practices—this has been accentuated as even more acute through a recent high-profile case of an older gay man in South Croydon receiving long-term homophobic treatment in a care home.^16^ Campaigning organisations in the UK have also recently released anecdotal reports of homophobic and transphobic treatment in care homes for older people.^17^

An additional issue is the lack of understanding of diversity and heterogeneity among older LGBTQ+ people as a cohort. There is expanding recognition of the ways in which gender-based differences manifest in different concerns and care needs for older lesbians^3 18^ and older trans people.^14 19^ Although there is a growing evidence base documenting the experiences and concerns of LGBTQ+ people about care provision in older age,^20^ a gap remains in evidence showing what works intervention-wise for enhancing care workers’ and providers’ knowledge and for developing more affirming staff attitudes and inclusive services.

The objective of this scoping review was to systematically review the current published and grey literature on interventions targeted at care staff members’ views and attitudes of LGBTQ+ older people in care and their needs. The review will identify gaps in knowledge to inform the development of a theory of change and identify potential interventions, which could address a change in views and attitudes of this population by care staff members. A preliminary search of PROSPERO, Medline, the Cochrane Database of Systematic Reviews and Joanna Briggs Institute (JBI) Evidence Synthesis was conducted and no current or in-progress scoping reviews or systematic reviews that focus on interventions relating to staff attitudes or behaviours of older LGBTQ+ people receiving social care were identified. This review is intended to inform future research ideas.

### Review questions

What interventions address care staff views, attitudes and knowledge of older LGBTQ+ people in residential and homecare settings?

Sub-questions to be addressed include:

1. What factors enable affirmative change in care staff members’ views, attitudes and knowledge about older LGBTQ+ people?
2. What factors pose as barriers to implement change in care staff members’ views, attitudes and knowledge about older LGBTQ+.

## Methods and analysis

This scoping review will be conducted following the JBI methodology for the conduct of scoping reviews^21^ and will be reported in line with the Preferred Reporting Items for Systematic Reviews and Meta-Analyses extension for Scoping Reviews (PRISMA-ScR) guidelines.^22^

### Inclusion and exclusion criteria

Inclusion and exclusion criteria have been developed in line with JBI methodology and categorised in terms of ‘Participant’, ‘Concept’ and ‘Context’. A summary of this is outlined in table 1 and more detail is described below.

**Table 1:** Inclusion and exclusion criteria

	Inclusion	Exclusion
Population	LGBTQ+ service users	Non-LGBTQ+ service users
	Older (>50 years) service users	Younger (<50 years) service users
	Receiving adult social care	Care not social care
Concept	Staff views/attitudes/knowledge	LGBTQ+ population views/experiences
	Interventions, ie, training/awareness raising/education, etc (face-to-face and online)	No intervention reported
	Care staff, ie, nurses, domiciliary care	does not include care staff
Context	Broad healthcare settings (including community), ie, residential care, care homes, hospitals, homes, etc	Non-relevant care settings

LGBTQ+lesbian, gay, bisexual, trans and queer

### Types of sources

This scoping review will consider quantitative, qualitative and mixed methods study designs for inclusion. We will include peer-reviewed published and grey literature as well as PhD theses and we will hand search a list of key websites (ie, third sector) identified by the lead/subject expert. We will include systematic reviews and scoping reviews that report on potentially relevant studies to hand-search their reference lists and ensure no key studies have been missed. We will exclude any opinion papers, commentaries, blogs or conference proceedings.

### Search terms

In line with JBI methodology, search terms have been developed within the categories of ‘Participants’, ‘Concept’ and ‘Context’. Details of each are provided below:

#### Participants

This review will consider studies that include older LGBTQ+ populations who are receiving adult social care. We will include studies that report on participants older than 50 years. This age range has been broadened from the standard WHO definition of old age in line with international literature to capture studies reporting on an increasingly ageing population. Terms will be broken down to include a range of LGBTQ+ identities such as ‘gay’, ‘lesbian’, ‘trans’, etc. We will also include a range of terms to capture those older than 50 years, such as ‘old’, ‘elder’, etc. Papers that report on those aged under 50 will be excluded.

#### Concept

The concept of interest is interventions that address a change in views, attitudes or knowledge of staff. This includes interventions delivered both face-to-face and online. A range of terms will be used to capture possible descriptions of intervention types such as ‘training’, ‘awareness raining’ and ‘education’ as well as terms to capture a range of different staff ‘views’, ‘perceptions’ and ‘attitudes’. These terms must be used in relation to older LGBTQ+ people in care settings. Papers reporting on LGBTQ+ peoples’ experiences of receiving care will be excluded.

#### Context

The context will be where studies report on interventions which are being delivered within a broader healthcare setting and will not only be limited to residential or homecare settings (ie, hospitals) by a range of staff types or countries. To capture this, we will include terms of different staff types which will not only be limited to care staff such as ‘nurs*’, ‘health professional’, etc to capture a broad range of potentially eligible intervention types.

### Search strategy

The search strategy will follow the JBI four-stepped approach:

1. A preliminary search of PsycINFO and ASSIA was conducted to identify key terms and further inform our search strategy with input from a subject librarian. Details of search terms to be used are outlined in table 2.
2. Following this, we will conduct a comprehensive search using all identified key terms, adapted to each database. Databases to be searched include Social Services Abstracts (via ProQuest), Social Care online (via social care institute for excellence), Sociological Abstracts (via ProQuest), PsycINFO (via Ovid), Medline (via Ovid), CINAHL (via EBSCO), Scopus (via Elsevier), ASSIA (via ProQuest) and Web of Science.
3. We will then search any preidentified websites (ie, third sector, government, etc.) which may have relevant grey literature. This includes Opening Doors, LGBT Foundation, Skills for Care, the UK Government, Social Care Institute for Excellence and the National Institute for Health and Care Excellence.
4. Finally, we will hand-search reference lists of any systematic reviews included to identify any potentially relevant papers missed through the database search.

**Table 2:** Search terms according to ‘Population’, ‘Concept’ and ‘Context’

Search no.	Domain	Search terms
**1**	**Population 1**	“LGBT*” OR “Trans” OR “Gay” OR “Lesbian” OR “Bisexual” OR “Queer”
**2**	**Population 2**	‘‘Old*’’ OR ‘‘Elder*’’ OR ‘‘Adult’’ OR ‘‘Age’’
**3**	**Concept 1**	‘‘View’’ OR ‘‘Attitude’’ OR ‘‘Perception’’ OR ‘‘Knowledge’’ OR ‘‘Belief’’
**4**	**Concept 2**	‘‘Training’’ OR ‘‘Awareness raising’’ OR ‘‘Education’’ OR ‘‘Inclusivity’’ OR ‘‘Behavior change’’ OR ‘‘Behaviour change’’ OR ‘‘Panels’’ OR ‘‘Best practice’’ OR ‘‘Support’’ OR ‘‘Beliefs’’ OR ‘‘Assessment’’ OR ‘‘Audit’’
**5**	**Context**	‘‘Staff’’ OR ‘‘Care*’’ OR ‘‘Nurs*’’ OR ‘‘Health professional’’ OR ‘‘Manager’’

LGBTQlesbian, gay, bisexual, trans and queer

All international literature will be included, and where required foreign language papers will be translated using Google Translate to determine inclusion or exclusion. The search will be conducted for the past 20 years (ie, 1 January 2003 to 31 December 2023), due to the lack of evidence in this field before this time. A final search time limit is in place for practical reasons; however, before finalising data extraction and analysis, a search will be conducted from 2023 onwards to check for any new publications since the search was conducted. The search strategy has been designed according to the PRESS checklist.^23^

### Study/source of evidence selection

Following the search, all citations will be imported into software management tools (ie, Rayyan^24^ and Mendeley^25^) and all duplicates will be removed. Supported through Rayyan^24^ and following a pilot test, screening will be conducted by two independent reviewers and assessed against the inclusion criteria. We will first screen all papers by titles followed by abstracts. Where there are any disagreements, these will be discussed until agreement is reached. Where this is not possible, a third independent reviewer with subject expertise will be invited to screen the citation. All papers included following abstract screening will be accessed in full. Where these are not available, these will be sought through the support of the University intra-library loan system. Full texts will then be reviewed in detail by two or more reviewers against the inclusion criteria. Any foreign language citations included will be translated to assess against the inclusion criteria. Where a paper is deemed to not meet the inclusion criteria, this will be discussed by the reviewers until an agreement is reached or by involving a third reviewer. Reasons for exclusion will be recorded and reported in the review. The results of the search will be reported in full in the final scoping review and presented in a PRISMA-ScR flow diagram.^22^

### Data extraction

Data extraction will be conducted on all included papers using a tailor-developed extraction template. Three reviewers will conduct extraction and will initially pilot the extraction template using a few papers to assess the suitability of the template any required changes. Data extracted will include details regarding the participants, concept, context, study methods, country/location of intervention and key findings and where relevant any facilitators or barriers of the intervention as well as characteristics of each paper, that is, authors, journal, year of publication, etc. An extraction template will be drafted. Key items to extract will include Author name; Publication year; Journal/Publisher; Country; Language; Method/s/Design; Participant populations (ie, Type, Sample size and Age); Concept (intervention); Context (setting); Key findings and Strength & limitations. Where any amendments are required to the template, these will be discussed and implemented accordingly. Where reviewers do not agree, these changes will be assessed by an independent reviewer with subject expertise to reach a final decision. Any changes made will be detailed in the full scoping review findings. Where required corresponding authors of papers will be contacted for missing or additional data. Inline with JBI methodology,^21^ included papers will not be quality appraised due to the objective of the scoping review being on mapping the evidence-based with the aim of developing a theory of change.

### Data analysis and presentation

Extracted data will be presented in summarised table and will be coded using Thematic Analysis.^26^ Data will be summarised by types of interventions (ie, concept), population type and setting/staff type (ie, context) to help identify what types of interventions work in which type of setting for which types of people. Where relevant, we will additionally code data to identify specific barriers and facilitators of each intervention/setting type. Findings will then be summarised to identify gaps in knowledge and develop a theory of change. This in turn will help inform future research ideas.

### Patient and public involvement

No patient or public involvement is required for the work as this is a review of preexisting literature.

### Ethics and dissemination

Ethical approval is not required as no primary data are being collected. The findings from the review will be submitted for publication in a peer-reviewed journal for consideration. Findings will be shared with key stakeholders and policy-makers to support future prioritisation and policy level decision-making. To enable this, the authors have already made links with key individuals and raised awareness of this work. The authors plan to work closely with stakeholders to support development of future action plans.
