# Supplementary material for: Interventions addressing care staff views of older LGBTQ+ people in residential and homecare settings: a scoping review protocol
Source: BMJ Open. 2024 Oct 23;14(10):e086497. doi: 10.1136/bmjopen-2024-086497 (PMC11499820; doi:10.1136/bmjopen-2024-086497)
Supplement: Supplementary file 1 [file bmjopen-2024-086497.reviewer_comments.pdf]

PEER REVIEW HISTORY

BMJ Open publishes all reviews undertaken for accepted manuscripts. Reviewers are asked to complete a checklist review form and are provided with free text boxes to elaborate on their assessment. These free text comments are reproduced below.

ARTICLE DETAILS

Title (Provisional)

Interventions addressing care staff views of older LGBTQ+ people in residential and homecare settings: A Scoping Review protocol

Authors

Moriarty, Yvonne; Willis, Paul

VERSION 1 - REVIEW

**Reviewer**	**1**
**Name**	**Goodyear, Trevor**
**Affiliation**	**The University of British Columbia**
**Date**	**25-Mar-2024**
**COI**	**None**

Thank you for the opportunity to review this protocol article, bmjopen-2024-086497: “Interventions addressing care staff views of older LGBTQ+ people in residential and homecare settings: A Scoping Review protocol.” This study will scope two decades of peer-reviewed and grey literature on interventions targeted at care staff members’ views and attitudes of LGBTQ+ older people in care and their needs. The authors make a compelling case for this scoping review study, which is well positioned to address important gaps in knowledge and practice with this under-served population. The protocol presented is clear and methodologically sound, though there are some areas for improvement and/or further clarification. Below, I offer some suggestions for strengthening this manuscript and scoping review study:

1. Abstract: Replace “their sexuality” in the first sentence with “their sexual and gender identities,” or similar, to also include a focus on gender.

2. Introduction: This section is strong – well done. The sentence beginning with “This partly stems from…” was lengthy and hard to follow, and could be broken into two sentences; here, I also suggest writing “non-heterosexual and trans identities” in place of “homosexual and trans identities.” Including a brief definition of “adult social care” could help to better orient the reader. Similarly, the authors could specify what they conceptualize as “older” – I see

that this information is included in the methods but providing a rationale and/or citation to support this definition would strengthen the study.

3. Methods – type of sources: Consider including scoping reviews, in addition to systematic reviews, for hand searching and identification of pertinent citations for this review.

4. Methods – search strategy: If changes can still be made to the search strategy, I suggest revising and expanding the terms for LGBTQ+ people. This systematic review article may offer helpful guidance: https://doi.org/10.1371/journal.pone.0156210 . For example, “sexual and gender minority” and “sexual and gender minorities” are common terms that should be included. Additionally, “trans” is likely too broad and should be specified (e.g., trans person*, trans m*n, trans wom*n, transgender*, transexual*). The search terms “old” and “age” for Population 2 (p.7) also seem too broad for use on their own.

5. Methods – inclusion/exclusion criteria: The inclusion/exclusion criteria are not explicitly stated, and this makes it challenging to determine how the reviewers will screen the studies for relevance and final inclusion in their scoping review.

6. Methods – data analysis and presentation: Consider briefly identifying a guiding approach (and perhaps a supporting citation) for the narrative description to be provided for the extracted data. As one example, content analysis is used in many scoping reviews.

7. Ethics and dissemination: This section could be strengthened with attention to how the authors’ scoping review will inform policy, practice, and research, and/or how they plan to share the scoping review findings beyond the context of a peer-reviewed publication. I understand this is a protocol article, but brief acknowledgment (even a sentence or two) of study significance and knowledge translation would help to strengthen this article.

I hope the authors find these comments helpful. Thank you for your important work in this area.

**Reviewer**	**2**
**Name**	**Van de Velde, Dominique**
**Affiliation**	**Ghent University, occupational therapy**
**Date**	**08-May-2024**
**COI**	**i have no competing interests**

Dear authors

this is an interesting topic and a topic that really needs more research.

The socpign review that is proposed in this paper is weeldesigned, I have a few minor issues.

Page 6: why is there in the 'subquestion to be addressed' only LGBT people described, and not LGTBQ+?

I also wonder whether there is some bias in this question, you assume that there is a need for a change in care staff members' views, attitudes and knowledge. Is this a fact?

Small mistake: awareness raining on page 7 line 141.

page 9 line 188. About the data extraction, it should be specified beforehand how many reviewers will pilot the extraction template.

Line 193 page 9: Where reviewers do not agree, these changes will be assessed by an independent reviewer to reach a final decision. It should be clarified whether these reviewers have prior knowledge on the topic, are they experts, have they been selected based on their knowledge on LGTBQ+ or specifically on their scientific knowledge (without being biased on their prior knowledge).

I'm looking forward for the results of the actual scoping review.

Good luck with the study.

VERSION 1 - AUTHOR RESPONSE

Please see attached response letter.

Reviewer	Comment	Authors response
1	1.1 Abstract: Replace "their sexuality" in the first sentence with "their sexual and gender identities," or similar, to also include a focus on gender.	Thank you for this suggestion. We have amended as suggested.
	1.2.1Introduction: This section is strong – well done. The sentence beginning with "This partly stems from…" was lengthy and hard to follow, and could be broken into two sentences; here, I also suggest writing "non-heterosexual and trans identities" in place of "homosexual and trans identities." Including a brief definition of "adult social care" could help to better orient the reader.<br><br>1.2.2 Similarly, the authors could specify what they conceptualize as "older" – I see that this information is included in the methods but providing a rationale and/or citation to support this definition would strengthen the study.	Thank you for highlighting this. We agree this was difficult to follow and have broken the sentence down and amended the wording as suggested to make it easier to read.<br><br>1.2.2: Thank you for this point. We have added a few sentences in the introduction to address this and also expanded the methods to add a justification for the age rage chosen. In essence this was to be as inclusive as possible and based on a broadening of WHO definition of age in order to capture an

		increasingly aging population and associated health needs.
	1.3 Methods – type of sources: Consider including scoping reviews, in addition to systematic reviews, for hand searching and identification of pertinent citations for this review.	1.3 Thank you for spotting this error. We have added in inclusion of scoping reviews.
	1.4 Methods – search strategy: If changes can still be made to the search strategy, I suggest revising and expanding the terms for LGBTQ+ people. This systematic review article may offer helpful guidance: https://doi.org/10.1371/journal.pone.0156210 . For example, "sexual and gender minority" and "sexual and gender minorities" are common terms that should be included. Additionally, "trans" is likely too broad and should be specified (e.g., trans person*, trans m*n, trans wom*n, transgender*, transexual*). The search terms "old" and "age" for Population 2 (p.7) also seem too broad for use on their own.	1.4 Thank you for this suggestion. Unfortunately the searches have already been carried out and it is not possible to update the search terms at this point. We can confirm that where we have used broad terms such as old* and trans* we will filter these through title and abstract screening and only include those that meet the inclusion criteria.
	1.5 Methods – inclusion/exclusion criteria: The inclusion/exclusion criteria are not explicitly stated, and this makes it challenging to determine how the reviewers will screen the studies for relevance and final inclusion in their scoping review.	1.5 Thank you for highlighting this. We have included a table to help clarify the inclusion/ exclusion criteria according to the JBI categories of Population, Concept and Context. We hope this makes it easier to follow.
	1.6 Methods – data analysis and presentation: Consider briefly identifying a guiding approach (and perhaps a supporting citation) for the narrative description to be provided for the extracted data. As one example, content analysis is used in many scoping reviews.	1.6 Thank you for this suggestion. We have rephrased this to include using Thematic Analysis.
	1.7 Ethics and dissemination: This section could be strengthened with attention to how the authors' scoping review will inform policy, practice, and research, and/or how they plan to share the scoping review findings beyond the context of a peer-reviewed publication. I understand this is a protocol article, but brief acknowledgment (even a sentence or two) of study significance and knowledge translation would help to strengthen this article.	1.7 Thank you for this suggestion. We have added a few sentences to highlighting that findings will be shared with key stakeholders and will support future policy decision making.

2	2.1.1 Page 6: why is there in the 'subquestion to be addressed' only LGBT people described, and not LGTBQ+?<br><br>2.1.2 I also wonder whether there is some bias in this question, you assume that there is a need for a change in care staff members' views, attitudes and knowledge. Is this a fact?	2.1.1 Apologies for this error, we have now corrected this.<br><br>2.1.2 Thank you for raising his point. This question has arisen from one of the authors previous work and the wider literature, from which we know that this is an area which is currently lacking research and needs more exploring.
	2.2 Small mistake: awareness raining on page 7 line 141.	2.2 Thank you for spotting this typo, we have corrected this.
	2.3 page 9 line 188. About the data extraction, it should be specified beforehand how many reviewers will pilot the extraction template.	2.3 Thank you for highlighting this. We have corrected this from 'two or more' to 'three' reviewers.
	2.4 Line 193 page 9: Where reviewers do not agree, these changes will be assessed by an independent reviewer to reach a final decision. It should be clarified whether these reviewers have prior knowledge on the topic, are they experts, have they been selected based on their knowledge on LGTBQ+ or specifically on their scientific knowledge (without being biased on their prior knowledge).	2.4 Thank you for highlighting this. We have added a few words to both the data extraction and screening section to outline that the independent reviewer has subject expertise in this area.<br><br>We would like to note that at the time of drafting the protocol it was not clear exactly who the other reviewers would be. It is therefore not appropriate to comment on the reviewers experience levels in this manuscript, however we will include this detail when drafting the results manuscript.

VERSION 2 - REVIEW

**Reviewer**	**1**
**Name**	**Goodyear, Trevor**
**Affiliation**	**The University of British Columbia**
**Date**	**10-Jul-2024**
**COI**	**None**

Thank you to the authors for their work on revising this scoping review protocol paper. The changes made have helped to strengthen and clarify the work, and I have no further comments. The added inclusion/exclusion criteria table is especially helpful. Well done!

Reviewer 3

Name Higgins , Agnes

Affiliation School of Nursing and Midwifery, Trinity College Dublin

Date 16-Aug-2024

COI I have no competing interests

Thank you for addressing the comments, I have some minor issues that still need to be addressed.

1. Abstract: The stated objective in the abstract appears to be different to the protocol, please relook and clarify.

2. In the inclusion/exclusion table

A) the symbols used for greater and lesser appear to be in the wrong place

B) Are both face to face and online interventions being included? This needs to be made explicit in the inclusion/exclusion table.

3. In the section on data extraction and data analysis you mention barriers to implementation, yet your review question only mentions enablers to change. Can you relook at this as well and clarify.

4. State not going to assess quality of the papers, you need to write a line to say why quality of the studies not being assessed, as surely quality impacts the robustness of outcomes and conclusions of the included studies.

VERSION 2 - AUTHOR RESPONSE

Reviewer	Comment	Authors response
Editor	Please include in your text the rationale for your assumption that there is a need for a change in care staff members' views, attitudes and knowledge.	Thank you for this suggestion. We have added a few additional sentences in the introduction to help clarify this. (line 96-100)
	Please provide a rationale for ending your search in 2023	Thank you for this query. The reason for the end date was due to pragmatic reasons as the search was due to be carried out in early 2023, and therefor was unlikely to bring up any new papers in that

		period. Prior to finalising the data extraction and analysis, we will do a final search from 2023 onwards to ensure nothing new has been published in that time. We have added information on this (line 182-184).
	You may note there is an extra reviewer on this revision who did not review the previous version of the manuscript. I apologise but there was a clerical error and an extra reviewer was invited when we would normally only invite the previous reviewers. However we believe the reviewer provided useful comments which would improve the protocol so we decided to include the comments in this letter.	Thank you for highlighting this. We have addressed the additional reviewers comments and provided responses to each of the points below. We agree these comments have improved our manuscript.
1	Thank you to the authors for their work on revising this scoping review protocol paper. The changes made have helped to strengthen and clarify the work, and I have no further comments. The added inclusion/exclusion criteria table is especially helpful. Well done!	Thank you for your invaluable comments, they have significantly improved the review methods and paper.
3	1. Abstract: The stated objective in the abstract appears to be different to the protocol, please relook and clarify.	Thank you for highlighting this to us. We have amended this in the abstract (line 36-38).
	2. In the inclusion/exclusion table<br>A) the symbols used for greater and lesser appear to be in the wrong place<br>B) Are both face to face and online interventions being included? This needs to be made explicit in the inclusion/exclusion table.	A) Thank you for noticing this error. This has been corrected in table 1.<br>B) We have added this in table 1 and also a sentence to the 'concept' section to clarify this (line 154).
	3. In the section on data extraction and data analysis you mention barriers to implementation, yet your review question only mentions enablers to change. Can you relook at this as well and clarify.	Thank you for noticing this error. This was an oversight. We have added a sub-question to our review questions (line 123-124).
	4. State not going to assess quality of the papers, you need to write a line to say why quality of the studies not being assessed, as surely quality impacts the robustness of outcomes and conclusions of the included studies.	Thank you for highlighting this. We are not conducting quality appraisal of included papers as the focus is on mapping the evidence to develop a theory of change. This is in line with JBI scoping review methods (see references). To make this clear we have added a sentence to the manuscript (line 218-220).

		Peters MDJ, Godfrey C, McInerney P, *et al*. Chapter 11: Scoping Reviews (2020 version). Aromataris E, Munn Z, editors. *JBI Manual for Evidence Synthesis*. *JBI*. 2020
